# *MYC* Gene Delivery to Adult Mouse Utricles Stimulates Proliferation of Postmitotic Supporting Cells *In Vitro*

**DOI:** 10.1371/journal.pone.0048704

**Published:** 2012-10-30

**Authors:** Joseph C. Burns, James J. Yoo, Anthony Atala, John D. Jackson

**Affiliations:** Wake Forest Institute for Regenerative Medicine, Wake Forest School of Medicine, Winston-Salem, North Carolina, United States of America; Instituto de Medicina Molecular, Portugal

## Abstract

The inner ears of adult humans and other mammals possess a limited capacity for regenerating sensory hair cells, which can lead to permanent auditory and vestibular deficits. During development and regeneration, undifferentiated supporting cells within inner ear sensory epithelia can self-renew and give rise to new hair cells; however, these otic progenitors become depleted postnatally. Therefore, reprogramming differentiated supporting cells into otic progenitors is a potential strategy for restoring regenerative potential to the ear. Transient expression of the induced pluripotency transcription factors, Oct3/4, Klf4, Sox2, and c-Myc reprograms fibroblasts into neural progenitors under neural-promoting culture conditions, so as a first step, we explored whether ectopic expression of these factors can reverse supporting cell quiescence in whole organ cultures of adult mouse utricles. Co-infection of utricles with adenoviral vectors separately encoding Oct3/4, Klf4, Sox2, and the degradation-resistant T58A mutant of c-Myc (c-MycT58A) triggered significant levels of supporting cell S-phase entry as assessed by continuous BrdU labeling. Of the four factors, c-MycT58A alone was both necessary and sufficient for the proliferative response. The number of BrdU-labeled cells plateaued between 5–7 days after infection, and then decreased ∼60% by 3 weeks, as many cycling cells appeared to enter apoptosis. Switching to differentiation-promoting culture medium at 5 days after ectopic expression of c-MycT58A temporarily attenuated the loss of BrdU-labeled cells and accompanied a very modest but significant expansion of the sensory epithelium. A small number of the proliferating cells in these cultures labeled for the hair cell marker, myosin VIIA, suggesting they had begun differentiating towards a hair cell fate. The results indicate that ectopic expression of c-MycT58A in combination with methods for promoting cell survival and differentiation may restore regenerative potential to supporting cells within the adult mammalian inner ear.

## Introduction

The sensory epithelia within the inner ears of adult mammals and humans are highly differentiated, postmitotic, and regeneration deficient. Thus, the loss of sound- and acceleration-detecting hair cells from auditory or vestibular sensory epithelia leads to permanent hearing or balance impairments, respectively. In contrast, the less differentiated sensory epithelia within the inner ears of developing mice and non-mammals of all ages are capable of more significant hair cell regeneration after damage, and non-mammals can recover sensory function [1]–[5].

During sensory epithelial development and regeneration, cells that morphologically resemble supporting cells act as otic progenitors that can self-renew and give rise to new hair cells. *In vitro* and *in vivo* evidence suggests that the progressive, postnatal depletion of these progenitors, likely via terminal differentiation, limits regeneration in mammals [4], [6]–[19].

Ectopic, long-term expression of the four transcription factors, Oct3/4, Sox2, Klf4, and c-Myc reprograms isolated somatic cells into induced pluripotent stem cells (iPSCs) [20]–[23]. The initial stages of the reprogramming process result in a partially dedifferentiated, “pre-iPSC” state, and transient expression of the iPSC factors has recently been utilized to directly reprogram somatic cells into lineage-restricted, multipotent progenitor/stem cells [24]–[29]. Therefore, applying the iPSC reprogramming technology – typically used with isolated somatic cells – to intact inner ear organs may be a novel approach for dedifferentiating adult mammalian supporting cells while they remain *in situ*.

Here, we show that adenoviral-mediated expression of a degradation-resistant mutant form of the iPSC factor, c-Myc, induces robust S-phase entry of supporting cells in cultured utricles from adult mice. In contrast, supporting cells remained postmitotic after ectopic expression of the three other iPSC factors, Oct3/4, Klf4, and Sox2. We present evidence that at least a portion of the cells were able to progress into M-phase, and a small number of cells replicating their DNA were found 21 days post-virus (DPV); however, many of the cycling cells appeared to enter apoptosis between 7 and 14 DPV. Switching from growth medium to serum-free differentiation medium could prevent the loss of cycling cells, but the protective effect of serum deprivation was temporary and subsided by 14 DPV. Within the protective time window, a modest but significant increase in the area of the sensory epithelium was detected at 10 DPV, and a very small number of cells that had replicated their DNA labeled with antibodies to the hair cell marker myosin VIIA. These results provide evidence that ectopic expression of c-MycT58A in inner ear organs may restore proliferative plasticity to postmitotic supporting cells.

## Materials and Methods

### Animals and dissection of utricles

All animal work was approved by the Animal Care and Use Committee of Wake Forest University (protocol number: A11–222). Swiss Webster mice, adults of either sex (>6 weeks old) and timed-pregnant females, were obtained from Charles River (Wilmington, MA). Labyrinths were dissected from temporal bones in ice-cold DMEM/F-12 (Invitrogen, Carlsbad, CA), the utricles were isolated, and the roof, otoconia, and nerve were mechanically removed under aseptic conditions. The dissected organs contained the entire sensory epithelium, a small portion of the surrounding non-sensory epithelium, and the underlying stromal tissue.

### Organ culture and infection with adenoviral vectors

Adenoviruses containing vectors encoding Oct3/4, Klf4, Sox2, c-MycT58A, or GFP under the control of a cytomegalovirus (CMV) promoter were obtained from Stemgent (Cambridge, MA). To construct its adenoviruses, Stemgent uses the AdEasy adenoviral vector system, which allows for insertion of a promoter and gene of interest into the E1-, E3-deleted backbone of adenovirus serotype 5. The cDNA plasmids cloned into the viral genome are described in Stadfeldt et al. [30] and can be obtained from Addgene. The Addgene plasmid ID numbers and final concentration of adenoviruses (transduction units per mL, TU/mL) used for the co-infection experiments were: mouse Oct3/4 (ID: 19768; titer: 5×10^7^ TU/mL), mouse Klf4 (ID: 19770; titer: 2×10^8^ TU/mL), mouse Sox2 (ID: 19767, titer: 5×10^7^ TU/mL), and human c-Myc T58A mutant with a hemagglutinin (HA) tag (ID: 19769, titer: 2×10^8^ TU/mL). The stock adenovirus solution comes stored in a suspension buffer consisting of 25 mM Tris (pH 7.5), 2.5 mM MgCl_2_, and 1 M NaCl. Stemgent titers its adenovirus using the immunoassay titration method.

For organ culture and adenovirus infection, dissected utricles were adhered to glass-bottom dishes (Mat-Tek, Ashland, MA; two utricles per dish) coated with Cell-Tak (BD Biosciences, San Jose, CA) as described [15] and maintained at 37°C and 5% CO_2_ in 100 µL of growth medium consisting of DMEM/F12, 5% FBS (Invitrogen), 0.25 µg/mL Fungizone (Invitrogen), and 10 µg/mL ciprofloxacin (Bayer, Berlin, Germany). Utricles were allowed to stabilize in culture for 24 h, after which the growth medium was replaced with infection medium (DMEM/F12, 0.25 µg/mL Fungizone, and 10 µg/mL ciprofloxacin). Adenoviruses were then added at the indicated concentrations for 8 h. Following adenovirus washout, utricles were cultured in 2 mL of growth medium until fixation. To label cells in S-phase, BrdU (3 µg/mL, Sigma) was added for the periods indicated. In some instances, the growth medium was replaced with 2 mL of differentiation medium to promote hair cell differentiation. Differentiation medium was based on a described formula [31] and consisted of DMEM/F12, N2 supplement (Invitrogen), 0.25 µg/mL Fungizone, and 10 µg/mL ciprofloxacin.

### Immunocytochemistry

The following antibodies were used: rabbit anti-myosin VIIA (1∶200; Proteus Biosciences, Ramona, CA; # 25-6790) and mouse anti-myosin VIIA (1∶100; Developmental Studies Hybridoma Bank, Iowa City, Iowa; # MYO7A 138-1) to label hair cell soma; mouse anti-BrdU (1∶50; BD Biosciences; # 347580) to label cells that had incorporated BrdU during S-phase; rabbit anti-Ki67 (1∶200; Thermofisher Scientific, Kalamazoo, MI; # RM-9106-S0) to label cells in the active G1, S, G2, and M phases of the cell cycle; mouse anti-phospho-histone H3 (Ser10) (PH3-Ser10; 1∶200, Cell Signaling Technology, Danvers, MA; # 9706) to label cells in M phase; rabbit anti-Oct3/4 (1∶200; Santa Cruz Biotechnology, Santa Cruz, California; # SC-5279); mouse anti-Klf4 (1∶200; Abcam, Cambridge MA; # AB75486); rabbit anti-Sox2 (1∶200; Millipore, Billerica, MA; # AB5603); mouse anti-c-Myc (1∶200; Santa Cruz Biotechnology; # SC-40); mouse anti-HA (1∶200; Abcam; # AB18181); and rabbit anti-activated-caspase 3 to label cells undergoing apoptosis (1∶200; Abcam; # AB3623).

For immunocytochemistry, utricles were fixed in fresh 4% paraformaldehyde in phosphate-buffered saline (PBS) for 1 h at room temperature (RT). After fixation, specimens were washed in PBS then permeabilized and blocked for 1 h at RT in PBS with 0.2% Triton X-100 (PBS-T) and 10% normal goat serum (NGS; Invitrogen). Samples to be labeled with anti-BrdU were digested with DNAse I (0.5 kunitz/µL; Sigma) for 1 h at 37°C before adding the blocking solution. Samples were then incubated in the appropriate primary antibodies in PBS-T with 2% NGS overnight, followed by 3 rinses in PBS-T and labeling with AlexaFluor-conjugated secondary antibodies (1∶200, Invitrogen) in PBS-T for 3 h at RT. Where indicated, AlexaFluor-conjugated phalloidin (5 U/mL, Invitrogen) and/or DRAQ5 (1∶1000, Cell Signaling) were included with the secondary antibodies to detect F-actin and nuclei. Utricles were rinsed in PBS 3 times and mounted in SlowFade (Invitrogen). Specimens were imaged using a Zeiss LSM 510 confocal microscope.

### Quantification

To quantify the percentage of GFP-expressing cells in Ad.GFP-infected utricles, the number of GFP-positive/myosin VIIA-negative supporting cells and GFP-positive/myosin VIIA-positive hair cells in 50 µm ×50 µm regions were separately counted at nine different locations spaced along the anterior-posterior axis of the medial edge, striola, and lateral edge of each utricle. The average for the nine regions was computed and then divided by previous estimates of the mean density of supporting cells and hair cells in adult mouse utricles *in vivo* [4], [32]. For total BrdU counts and cell cycle phase analysis, all BrdU-, Ki67-, and PH3-Ser10-labeled nuclei in the sensory epithelium were manually counted for each utricle using the Cell Counter plugin in ImageJ (U.S. National Institutes of Health, Bethesda, MD). Macular area was measured by using ImageJ to trace the outline of the sensory epithelium in confocal images of utricles labeled with anti-myosin VIIA.

### Statistics

OriginPro 7.5 was used to conduct sigmoidal equation fits, Student's t-tests, and one-way or two-way ANOVAs followed by Tukey's Test of Multiple Comparisons (alpha level  = 0.05 in all cases). All descriptive statistics are presented as mean ± s.e.m. To calculate nonlinear least squares fits, OriginPro uses Levenberg-Marquardt chi-squared minimization with automatic parameter initialization. To fit equations to the data, 200 iterations of this minimization routine were performed. The following sigmoidal equation was used for the fits:
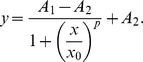



## Results

### Type 5 adenovirus transduces supporting cells and a small fraction of hair cells in adult mouse utricles *in vitro*

The inner ear sensory epithelium is difficult to transfect using techniques such as electroporation and lipofection [33]–[35]. Some viruses efficiently infect supporting cells and/or hair cells [36], [37], and ectopic gene expression in supporting cells has been observed after infecting adult mouse utricles with type 5 adenovirus *in vitro* [38]–[41]. To characterize the efficiency of ectopic expression versus adenovirus concentration, we infected utricles cultured from mice >6 weeks old with 10^6^–10^9^ transduction units per mL (TU/mL) of adenovirus that contained a vector encoding green fluorescent protein (Ad.GFP) under the control of a CMV promoter. The cultures were fixed at 3 days post virus (DPV), and the percentage of GFP-expressing supporting cells that did not label with antibodies for the hair cell marker myosin VIIA were quantified by sampling multiple regions within the sensory epithelium.

**Figure 1:**
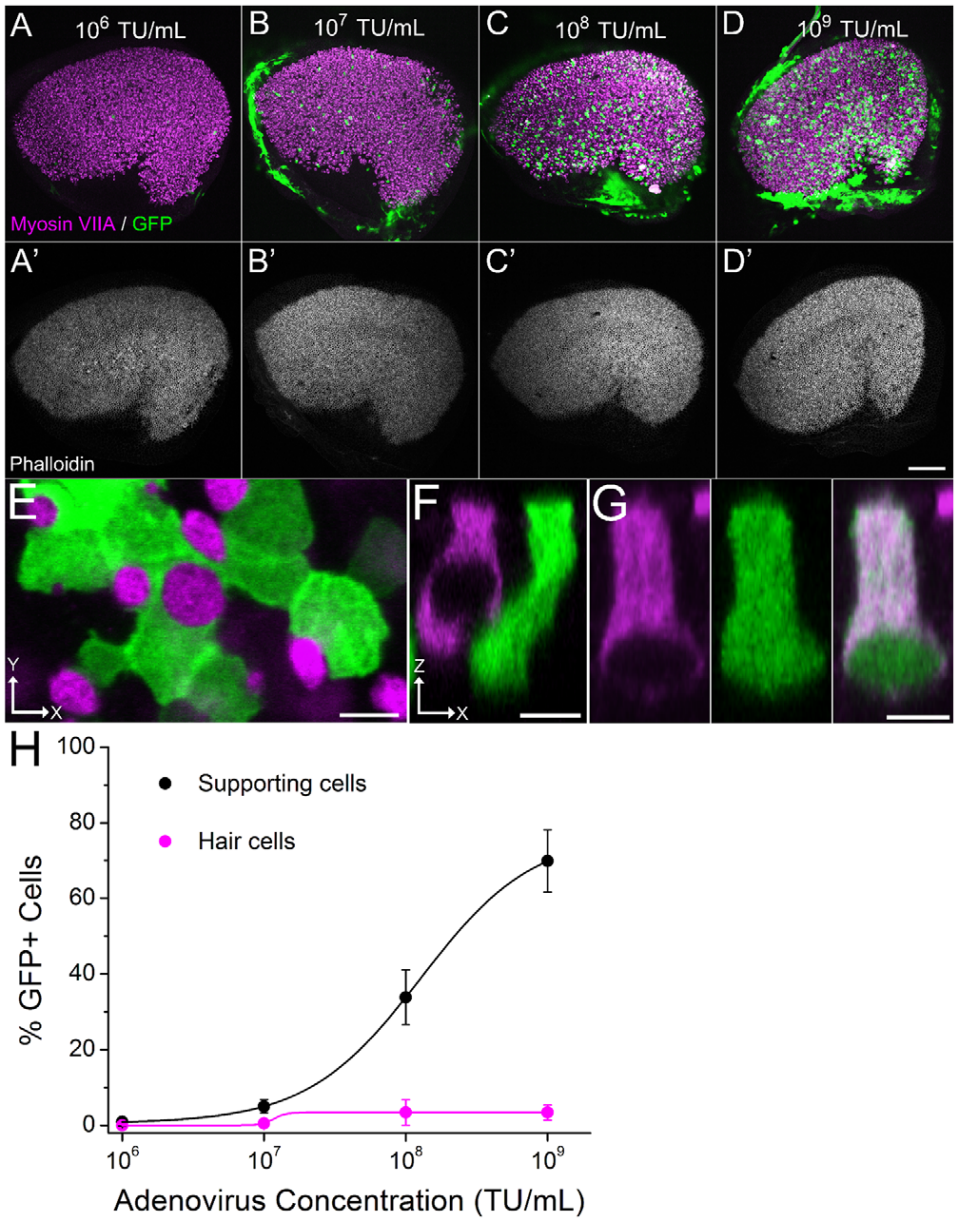
Adenovirus primarily infects supporting cells but also some hair cells in adult mouse utricles *in vitro*. (A–D) Low magnification (20×/0.75 NA) confocal images of utricles from adult mice that were incubated with increasing concentrations of adenovirus engineered to express green fluorescent protein (GFP, green) under the control of a CMV promoter. Utricles were fixed and labeled 3 d after adenovirus was washed out. Hair cells are labeled with antibodies to myosin VIIA (magenta). (A′–D′) Fluorescent phalloidin labeling in the utricles from A–D. Scale bar for A–D', 100 µm. (E) High-resolution (63×/1.4 NA) confocal section taken at the apical surface of the sensory epithelium shows a cluster of GFP-positive supporting cells (green). The supporting cell apical surfaces have characteristic polygonal shapes compared to the circular profile of hair cells labeled with anti-myosin-VIIA (magenta). (F) View of a confocal image stack parallel to the apical-basal axis of supporting cells and hair cells shows a supporting cell expressing GFP (green), but its neighboring myosin-VIIA-labeled hair cell (magenta) does not. Scale bar, 5 µm. (G) Same view as in F shows a GFP-expressing hair cell. Scale bar, 5 µm. (H) A graph showing quantification of the percentage of GFP-expressing supporting cells (black circles) and hair cells (magenta circles) with increasing concentration of adenovirus. Myosin VIIA labeling was used to distinguish between supporting cells and hair cells, and sigmoidal equations (black and magenta lines) were fit to the data points (see Table1 for equation and coefficients).

10^6^ TU/mL resulted in little to no GFP expression, but the percentage of supporting cells expressing GFP increased for concentrations ranging from 10^7^–10^9^ TU/mL (Fig. 1A–D, H; n = 2 utricles per concentration). Adenoviral-mediated GFP expression in utricular hair cells from neonatal mice has previously been modeled with a sigmoidal function [36]. Nonlinear least squares fitting to our data showed that the percentage of GFP-expressing supporting cells in adult mouse utricles could similarly be modeled with a sigmoidal function (r^2^ = 1), which had a half-maximal transduction efficiency at 1.3×10^8^ TU/mL and a maximum efficiency of 77.2% (Fig. 1H, Table 1). Within the range of Ad.GFP concentrations tested, the utricular macula maintained epithelial and cytoskeletal integrity as revealed with fluorescent phalloidin labeling (Fig. 1A'–D'). The GFP-expressing supporting cells displayed typical, cylindrical shapes that were more expanded at their apical and basal ends and compacted at the level of the hair cell nuclei (Fig. 1E–F).

**Table 1:** Coefficient values for sigmoidal curve fits.

	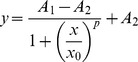			
	*A*_1_	*A*_2_	χ_0_	*p*
Supporting cells	0.5	77.2	1.3×10^8^	1.1
Hair cells	0	3.4	1.2×10^7^	10.6

Labeling of hair cells with antibodies to myosin VIIA allowed us to distinguish between hair cells and supporting cells, and we found that GFP expression was mostly restricted to supporting cells (Fig. 1E–F). However, we did find occasional GFP-positive/myosin VIIA-positive hair cells with characteristic flask-like shapes (Fig. 1G). The intensity of GFP-expression in these cells appeared weaker than in neighboring supporting cells, which may contribute to difficulty in identifying them since previous studies have reported that adenovirus type 5 exclusively expresses in supporting cells within adult mouse utricles [38]–[40]. The percentage of hair cells expressing GFP also fit with a sigmoidal function (r^2^ = 1) at virus concentrations ranging from 10^6^–10^9^ TU/mL, but the maximum hair cell transduction efficiency was only 3.4%, which was 23-times lower than for supporting cells (Fig. 1H, Table 1). This efficiency was also substantially lower than the ∼60% reported for hair cells in utricles from neonatal mice [36]. Also, the viral concentration yielding half-maximal hair cell transduction in adults was an order of magnitude lower than for supporting cells, but on the same order of magnitude for neonatal hair cells (Fig. 1H, Table 1; concentration at half-maximal infection  = 1.3×10^7^ TU/mL). Combined, the results suggest that adenovirus transduces hair cells at similar concentrations independent of age, the number of transduced hair cells decreases substantially with age, and adenovirus transduces adult hair cells at lower concentrations than adult supporting cells. It remains unclear whether the age-related decrease in GFP-expressing hair cells reflects a change in infectivity or CMV promoter activity. Although the number of GFP-expressing hair cells was relatively low, ectopic gene expression within hair cells may need to be taken into consideration when infecting adult mouse utricles with adenovirus *in vitro*.

### Adenoviral gene delivery of iPSC transcription factors to the adult mouse utricle initiates S-phase entry in postmitotic supporting cells

**Figure 2:**
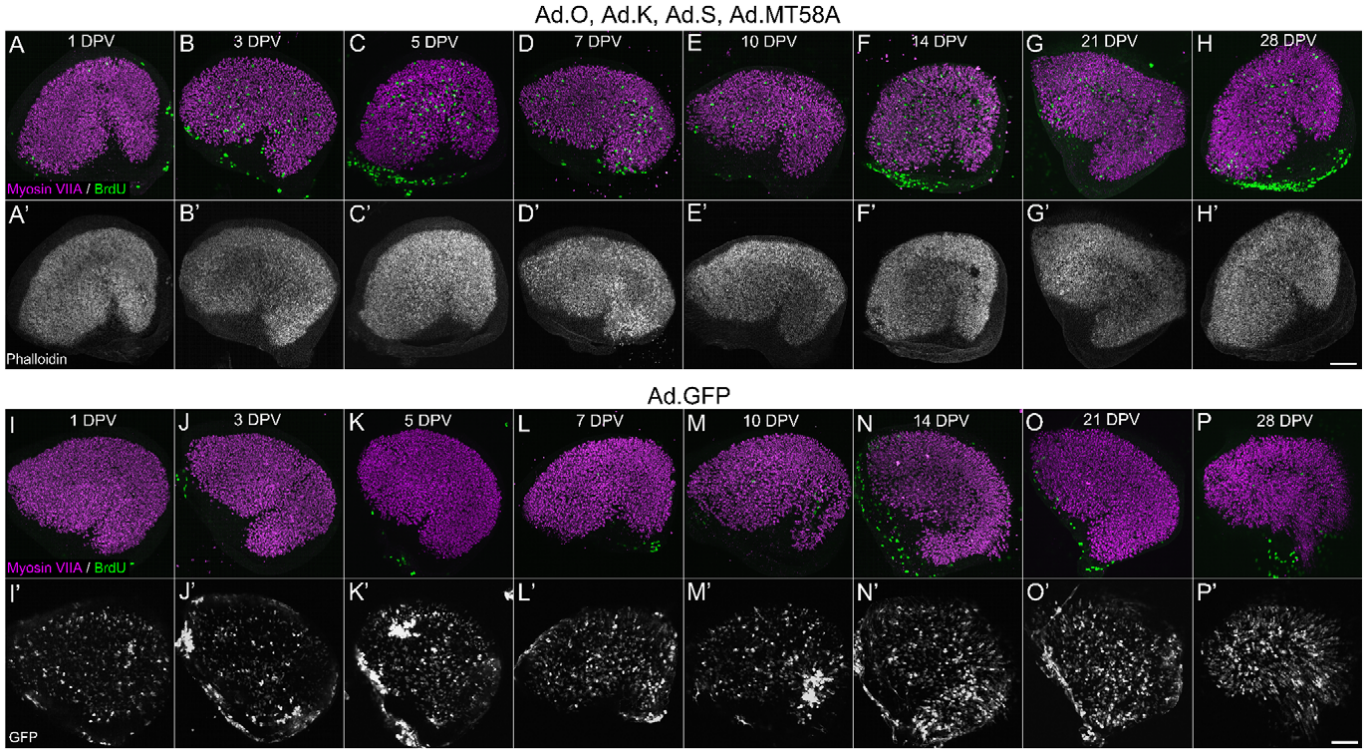
Co-infection of Ad.O, Ad.K, Ad.S, and Ad.MT58A induces cell cycle reentry of postmitotic supporting cells in adult mouse utricles *in vitro*. (A–F) Low magnification (20×/0.75 NA) confocal images of co-infected utricles from adult mice at various days post-virus (DPV). BrdU was included in the culture medium for the entire period after virus washout, and utricles were fixed and labeled at the times indicated. Hair cells and nuclei that entered S-phase are labeled with antibodies to myosin VIIA (magenta) and BrdU (green), respectively. (A'–F') Fluorescent phalloidin labeling in the utricles from A-F. Scale bar for A–F', 100 µm. (I–P) Low magnification (20×/0.75 NA) confocal images of control utricles infected with Ad.GFP. Few to no BrdU labeled nuclei are present in the sensory epithelium. (I'–P') GFP expression in the utricles from I–P. Scale bar for I–P', 100 µm.

After characterizing the adenoviral transduction efficiency, we next sought to determine whether ectopic expression of any of the four iPSC transcription factors could initiate cell cycle reentry of postmitotic supporting cells *in vitro*. For this, we co-infected adult mouse utricles with separate adenoviruses encoding mouse Oct3/4 (Ad.O, 5×10^7^ TU/mL), mouse Klf4 (Ad.K, 2×10^8^ TU/mL), mouse Sox2 (Ad.S, 5×10^7^ TU/mL), and the T58A mutant of human c-Myc (Ad.MT58A, 2×10^8^ TU/mL) under the control of CMV promoters and then added BrdU to the culture medium for the remainder of the culture period to label any cells that entered S-phase (n = 3–7 utricles per culture period). The T58A mutation confers resistance to degradation by preventing threonine phosphorylation [42]–[45]. For controls, we infected other utricles with Ad.GFP alone (5×10^8^ TU/mL; n = 2–5 utricles per culture period). Twenty-four h after washing out the virus, antibodies to BrdU did not label any nuclei in the sensory epithelium of utricles co-infected with Ad.O, Ad.K, Ad.S, and Ad.MT58A (Fig. 2A, I). However, by 3 DPV, the co-infected utricles contained many BrdU-positive nuclei in their sensory epithelia (Fig. 2B, J). We also found BrdU-positive nuclei in the maculae at six later timepoints ranging from 5 to 28 DPV (Fig. 2C–H). Induction of S-phase entry appeared to be specific to utricles co-infected with the four iPSC transcription factors since comparatively minimal BrdU labeling was detected in the sensory epithelium of Ad.GFP-infected utricles (Fig. 2I–P).

Even at 28 DPV, the sensory epithelium maintained its integrity and mitten-like shape, and supporting cells and hair cells maintained compaction with tall aspect ratios (Fig. 2A'–H'). Adenoviral vectors typically do not integrate into the host DNA, and they exhibit transient expression profiles in some cells, which may reduce the potential for tumorigenic effects from insertional mutagenesis or transformation [30]. Nevertheless, strong GFP expression persisted at qualitatively similar cellular densities for all 28 DPV (Fig. 2I'–P'). GFP expression also persisted in the surrounding non-sensory epithelium and underlying stromal tissue, suggesting that the replication-deficient adenovectors were not cleared or may have integrated into the host DNA. *LacZ* expression from adenoviral vectors has also been observed out to one month after delivery of adenovirus to mouse utricles *in vivo* [46].

**Figure 3:**
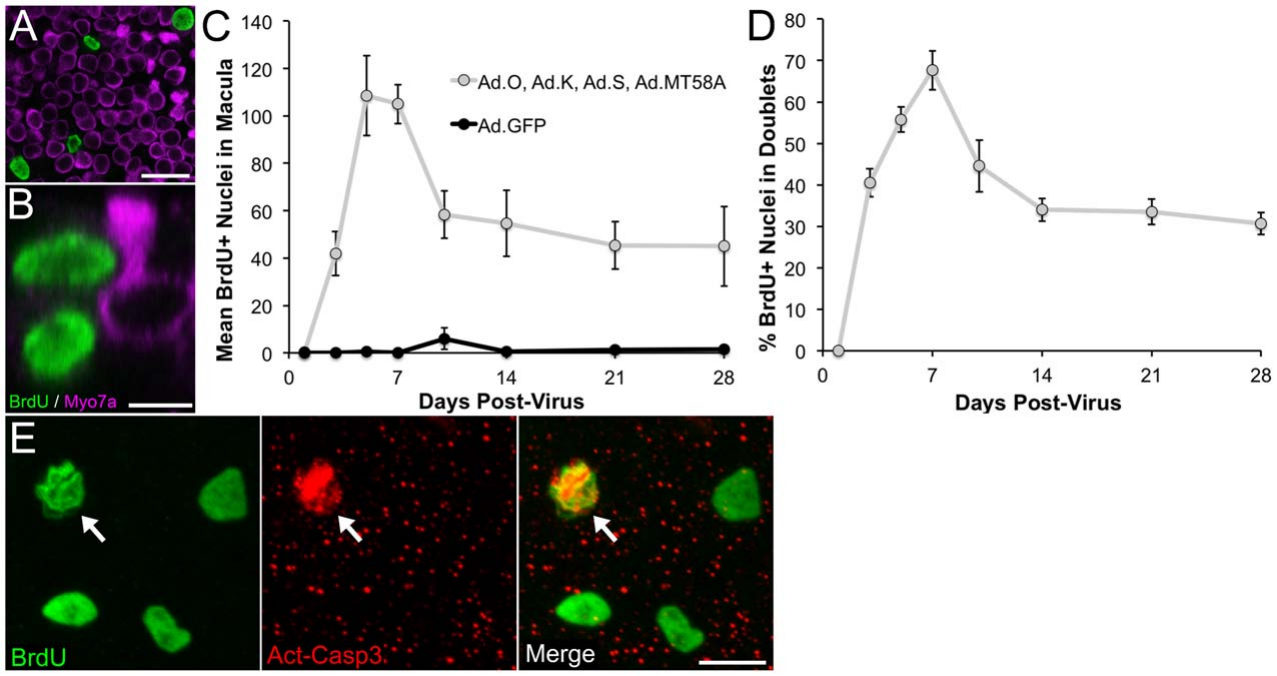
BrdU-labeled cells in utricles co-infected with Ad.O, Ad.K, Ad.S, and Ad.MT58A are present after four weeks in culture and many appear in doublets. (A–B) High-resolution (63×/1.4 NA) views both orthogonal (A) and parallel to the apical-basal axis of supporting cells and hair cells in a co-infected utricle that was cultured for 7 days post-virus. BrdU was included in the culture medium for the entire period after virus washout. Hair cells and nuclei that have entered S-phase are labeled with antibodies to myosin VIIA (magenta) and BrdU (green), respectively. The two BrdU-positive nuclei in B appear to be a division pair, with one positioned at the level of hair cell nuclei and the other at the supporting cell nuclear layer. Scale bar in A, 20 µm. Scale bar in B, 5 µm. (C) Graph shows quantification of the mean number of BrdU-positive nuclei per sensory epithelium versus time in culture. Data from co-infected utricles are shown in gray, and data from control utricles infected with GFP are shown in black. (D) Graph shows the percentage of BrdU-positive nuclei that appeared as doublets in co-infected utricles (same utricles used for the gray data points in C). Subtracting the percentage from 100 yields the percentage of nuclei that appeared as singlets. (E) Confocal image of a co-infected utricle fixed at 8 days post-virus and labeled with antibodies to BrdU (green) and activated caspase 3 (red). Arrow points to a pyknotic nucleus that labeled with both antibodies. Scale bar, 10 µm.

None of the BrdU-positive nuclei in the sensory epithelium labeled with antibodies to myosin VIIA, indicating that only supporting cells were entering S-phase and none of the labeled cells had differentiated into new hair cells when cultured with growth medium (Fig. 3A–B). Quantification of the mean number of BrdU-positive supporting cells revealed that S-phase entry peaked between 5 and 7 DPV (mean BrdU-positive nuclei at 7 DPV  = 105±8; Fig. 3C). The mean number of BrdU-labeled nuclei per sensory epithelium declined sharply by 45% from 7 to 10 DPV, and then decreased more gradually and eventually stabilized between 10 and 28 DPV (mean BrdU-positive nuclei at 28 DPV  = 45±17; Fig. 3C). Since BrdU was included in the culture medium throughout, a decline in the number of BrdU-positive nuclei indicates that the population of BrdU-labeled cells somehow became depleted, likely via cell death or exclusion from the sensory epithelium. Antibodies to activated caspase 3 co-labeled 2–3 BrdU-positive cells per sensory epithelium at 8 DPV (n = 2 utricles), suggesting that the depletion of BrdU-positive cells was due to apoptosis (Fig. 3E). The small number of activated-caspase-3-labeled cells may have been due to rapid apoptosis [39].

Many of the BrdU-labeled nuclei were present in pairs, triplets, or quadruplicates, suggesting that some cells were completing mitosis and dividing (Fig. 3A–B, D). Quantification showed that the percentage of BrdU-positive nuclei per sensory epithelium appearing in doublets also peaked between 5 and 7 DPV (Fig. 3D; percentage of doublets at 7 DPV  = 68±8%). The percentage of doublets declined thereafter, following a similar trend as the mean number of BrdU-labeled nuclei (Fig. 3D; percentage of doublets at 28 DPV  = 31±3%). Together, the results suggest that many cells were passing through S-phase, completing M phase and cytokinesis, and then dying. Since the levels of BrdU labeling eventually stabilized and some doublets were still detected at 28 DPV, a fraction of cells may be capable of surviving after induction of cell cycle reentry with iPSC transcription factors.

### Of the four iPSC transcription factors, c-MycT58A is both necessary and sufficient for inducing reentry of supporting cells into the cell cycle

**Figure 4:**
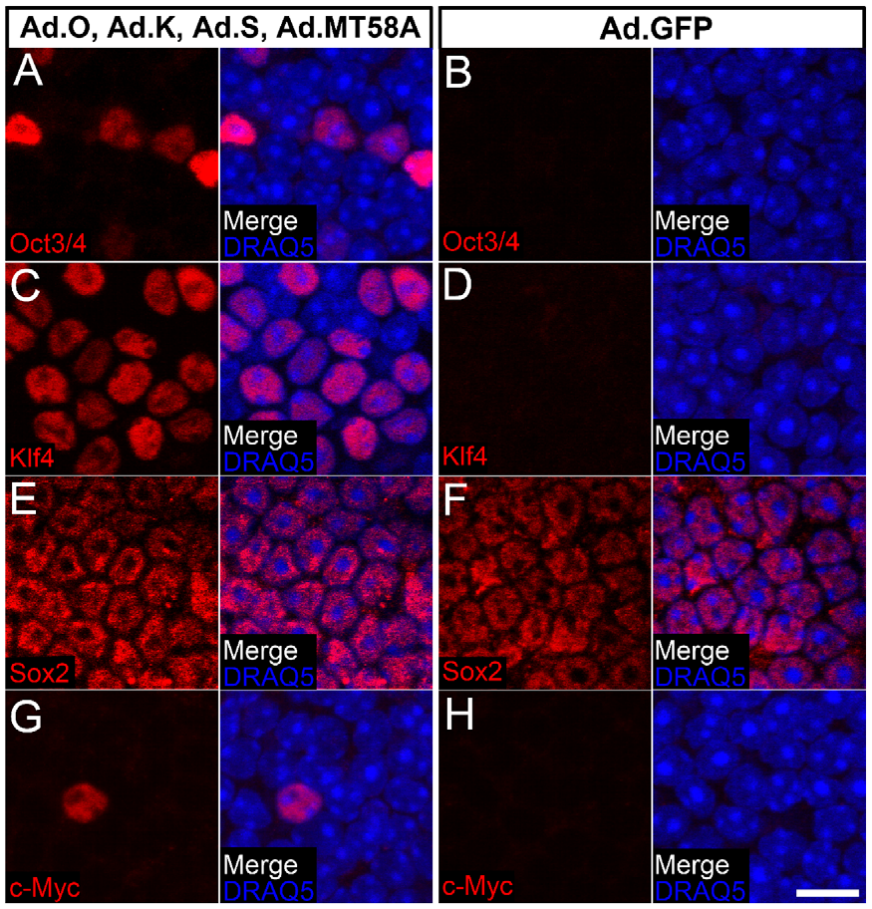
Infection with Ad.O, Ad.K, or Ad.MT58A leads to detectable increases in Oct3/4, Klf4, or c-Myc protein levels in some supporting cells. Utricles were separately infected with Ad.O, Ad.K, Ad.S, Ad.MT58A, or Ad.GFP (5×10^8^ TU/mL) and fixed at 3 days post-virus. Shown are single confocal slices (0.75 µm z-thickness) at the level of the supporting cell nuclei. Supporting cell nuclei are labeled with the DNA dye, DRAQ5 (blue). The same amplifier gain and offset settings used to acquire images of the treated samples were used for acquiring images of their respective Ad.GFP controls. Note the similar intensity of Sox2 labeling in utricles infected with Ad.S and Ad.GFP. Scale bar for A–H, 10 µm.

Antibody labeling of 3 DPV utricles that were separately infected with Ad.O, Ad.K, Ad.S, or Ad.MT58A (each at 5×10^8^ TU/mL) showed increased protein levels of Oct3/4, Klf4, and c-Myc in some supporting cell nuclei (Fig. 4A, C, G). Oct3/4, Klf4, and c-Myc antibody labeling was not detectable in utricles infected with Ad.GFP, which indicated that the increased protein levels were a result of ectopic expression from Ad.O, Ad.K, and Ad.MT58A, respectively (Fig. 4B, D, H). Sox2 is expressed in supporting cells and a subset of hair cells in the adult mouse utricle *in vivo* [47], and the intensity of Sox2 antibody labeling did not appear to differ between Ad.S- and Ad.GFP-infected utricles (Fig. 4E, F).

Antibodies to Klf4 labeled the greatest number of nuclei, whereas c-Myc antibodies labeled <10 nuclei per sensory epithelium (Fig. 4A, G). Ad.MT58A-infected utricles fixed and labeled with c-Myc antibodies at 1, 2, 5, and 10 DPV also contained similarly low numbers of c-Myc-positive supporting cells, as did antibody labeling for the hemagglutinin (HA) tag that was engineered into the c-MycT58A transgene (data not shown).

**Figure 5:**
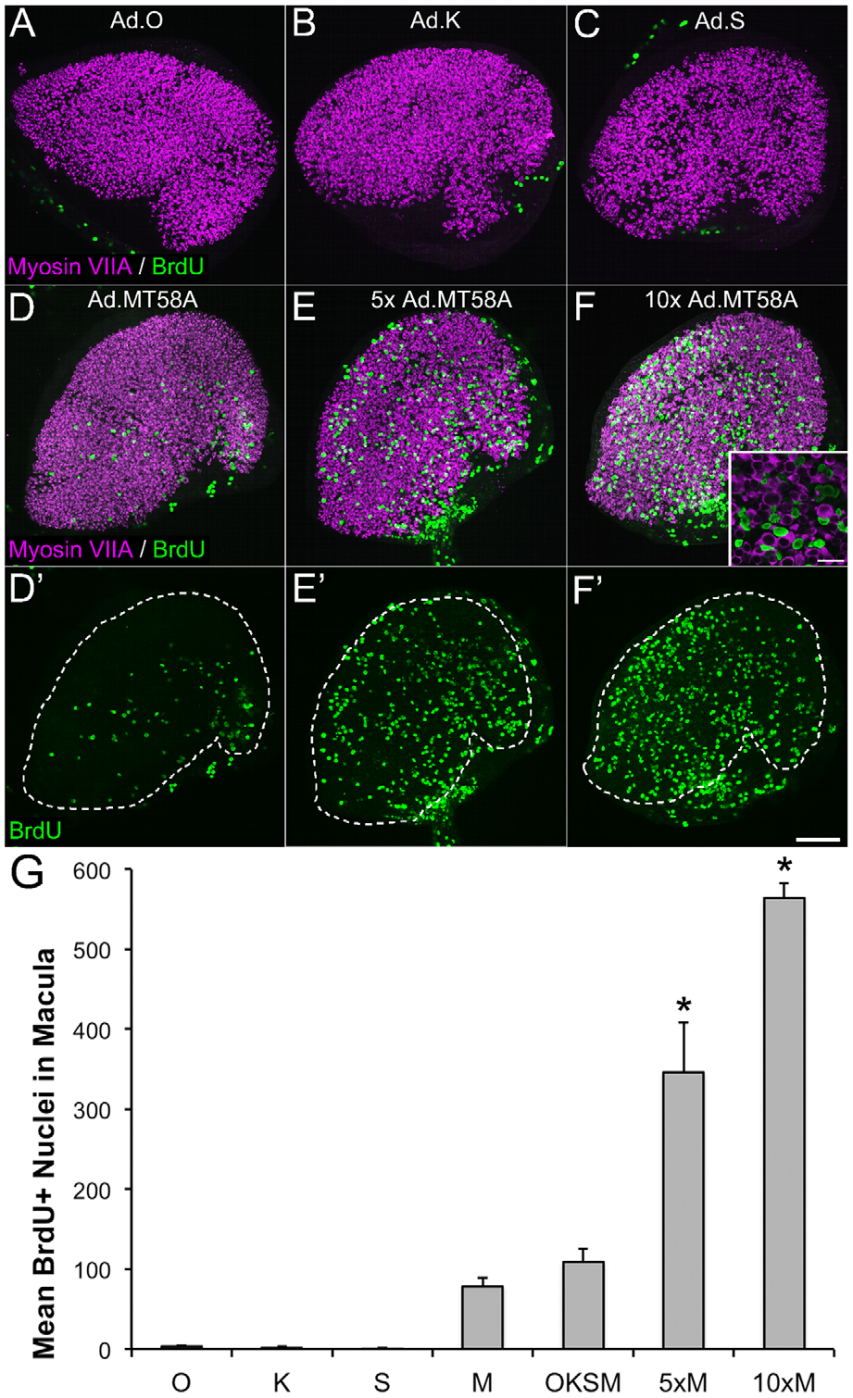
Infection with Ad.MT58A is both necessary and sufficient for the observed proliferative response. (**A–D**) Confocal images of utricles that were individually infected with Ad.O, Ad.K, Ad.S, or Ad.MT58A and cultured in the presence of BrdU for 5 days post-virus (adenovirus concentrations equal to those used for co-infection experiments). Significant BrdU labeling (green) is only detected in the sensory epithelium of a utricle infected with Ad.MT58A. Hair cells are labeled with an antibody to myosin VIIA (purple). (**E–F**) Confocal images of utricles infected with 1×10^9^ TU/mL (5×) and 2×10^9^ TU/mL (10x) of Ad.MT58A. Inset in F shows a zoomed region of the sensory epithelium. Scale bar for inset, 20 µm. (**D'–F'**) Confocal images of the BrdU channel in D–F without the myosin VIIA overlay. White dashed lines demarcate the borders of the sensory epithelium. Scale bar for A–F', 100 µm. (**G**) Quantification of the number of BrdU-labeled nuclei per sensory epithelium for the different adenovirus combinations tested. O: Ad.O, K: Ad.K, S: Ad.S, M: Ad.MT58A, OKSM = co-infection with Ad.O, Ad.K, Ad.S, and Ad.M T58A. The difference in the number of BrdU-positive nuclei in co-infected utricles and utricles infected with Ad.MT58A did not reach statistical significance, but the increases in BrdU-positive nuclei at higher concentrations of Ad.MT58A were significant (asterisks indicate a significant difference from all other conditions; p<0.05; One-way ANOVA with Tukey's Test of Multiple Comparisons; n = 4 utricles).

Since the ectopic protein levels appeared to differ for each factor, we next sought to determine whether cell cycle reentry was dependent on one individual factor by separately infecting adult mouse utricles with Ad.O (5×10^7^ TU/mL), Ad.K (2×10^8^ TU/mL), Ad.S (5×10^7^ TU/mL), or Ad.MT58A (2×10^8^ TU/mL) at viral concentrations identical to those used for the co-infection experiments. BrdU was included in the culture medium for the remainder after virus washout. Fixing the cultures at 5 DPV and labeling for BrdU revealed significant numbers of supporting cells that had reentered the cell cycle in utricles infected with Ad.MT58A compared to those infected with Ad.O, Ad.K, or Ad.S (Fig. 5A–D; mean BrdU-positive supporting cells in Ad.MT58A-infected utricles  = 79±10; p<0.05, One-way ANOVA with Tukey's Test of Multiple Comparisons; n = 9 utricles). Increasing the concentration of Ad.MT58A 5- or 10-times significantly enhanced the numbers of BrdU-labeled nuclei at 5 DPV (Fig. 5D–G; mean BrdU-positive supporting cells at 1×10^9^ TU/mL  = 357±65, mean BrdU-positive supporting cells at 2×10^9^ TU/mL  = 564±19; p<0.05, One-way ANOVA with Tukey's Test of Multiple Comparisons; n = 2–4 utricles), and increasing the concentration of Ad.O, Ad.K, or Ad.S to 1×10^9^ TU/mL did not result in significant S-phase entry (data not shown). Thus, of the four transcription factors, c-MycT58A appears to be both necessary and sufficient for the induction of cell cycle reentry.

The levels of S-phase entry in Ad.MT58A-infected (2×10^8^ TU/mL) utricles were similar to those in utricles co-infected with Ad.O (5×10^7^ TU/mL), Ad.K (2×10^8^ TU/mL), Ad.S (5×10^7^ TU/mL), and Ad.MT58A (2×10^8^ TU/mL; Figs. 3C, 5G). When we double-labeled Ad.MT58A-infected utricles with antibodies to c-Myc and Ki-67, a protein that is upregulated during the active phases of the cell cycle [48], we detected one nucleus in five specimens that labeled with both antibodies, indicating that few actively cycling cells had detectable levels of c-MycT58A protein. While there were relatively few c-Myc-positive cells compared to the large number of proliferating cells, it remains possible that autonomous expression of c-MycT58A in supporting cells induces cell cycle reentry, but c-MycT58A protein gets degraded to undetectable levels as the cells transition out of quiescence. Therefore, we were not able to determine whether cell cycle reentry is driven by cell autonomous expression of c-MycT58A or whether ectopic expression in neighboring cells stimulates the proliferative response via paracrine signaling.

### Supporting cells in Ad.MT58A-infected utricles can proceed to mitosis

Many of the BrdU-positive nuclei in utricles infected with just Ad.MT58A appeared in doublets (Fig. 5F, inset), suggesting these cells completed all phases of the cell cycle and divided. To further characterize the effects of Ad.MT58A infection on cell cycle progression in supporting cells, we co-labeled Ad.MT58A-infected utricles (1×10^9^ TU/mL) fixed at 5, 7, and 10 DPV with antibodies to BrdU and Ki-67. Since BrdU is permanently incorporated into DNA during replication in S-phase, cells that label with antibodies to both Ki67 and BrdU have replicated their DNA and are still actively cycling. Cells that label for just BrdU have replicated their DNA and exited the cell cycle. Cells that label for just Ki67 have not yet replicated their DNA and are presumed to be in G1.

**Figure 6:**
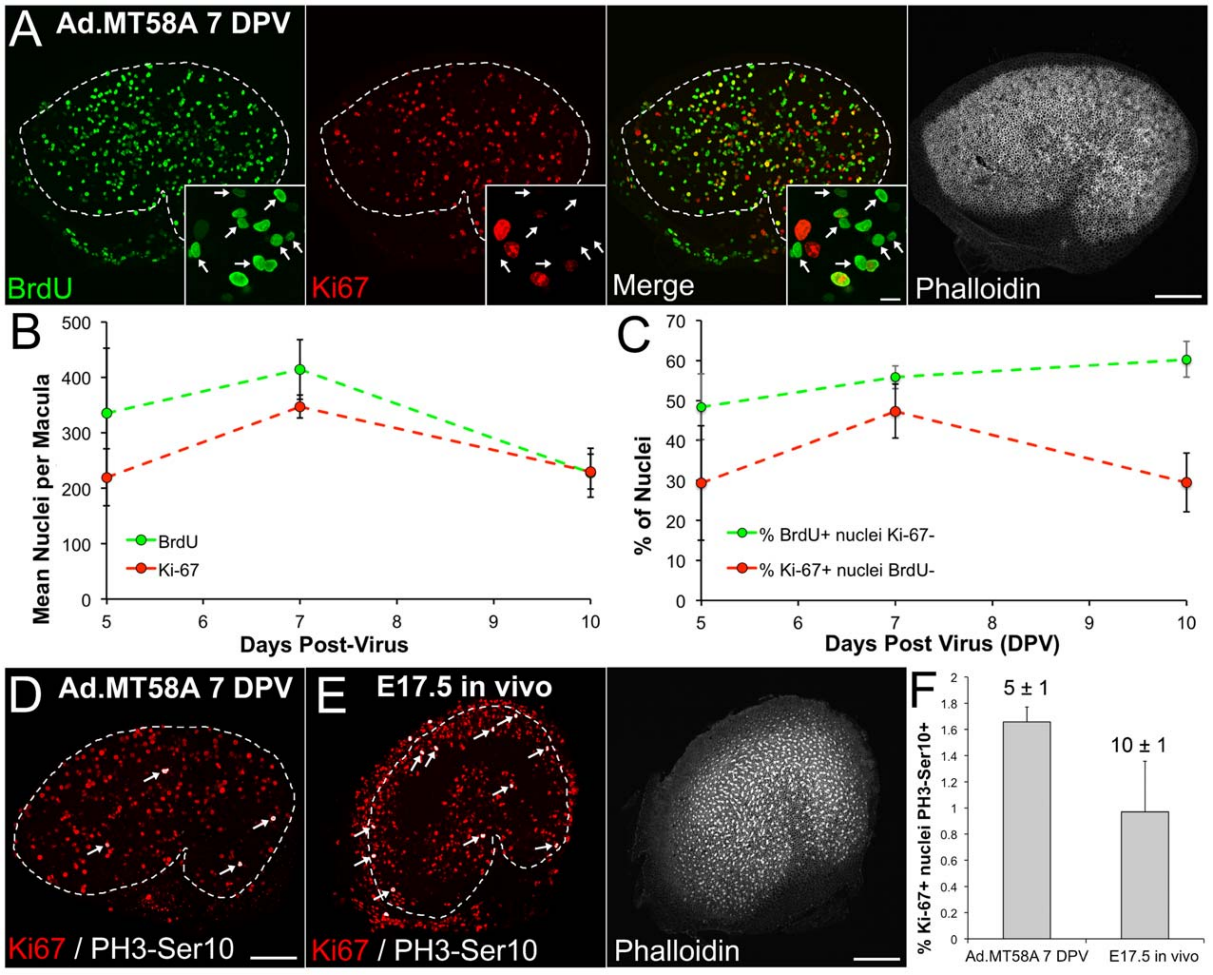
Supporting cells that reenter the cell cycle after Ad.MT58A infection can progress to mitosis. (**A**) Confocal images show an Ad.MT58A-infected utricle (1×10^9^ TU/mL) that was fixed at 7 days post-virus (DPV) and co-labeled with antibodies to BrdU (red) and Ki-67 (green). Phalloidin labeling (grayscale) is shown to aid in visualizing the borders of the sensory epithelium. White dashed lines demarcate the borders of the sensory epithelium. Scale bar, 100 µm. Insets show high-resolution views of nuclei in the sensory epithelium. Arrows indicate nuclei that labeled with antibodies to BrdU but not Ki67. Scale bar for insets, 5 µm. (**B**) Graph shows the mean number of BrdU-labeled nuclei (green data points) and Ki-67-labeled nuclei (red data points) per sensory epithelium at 5, 7, and 10 DPV. (**C**) Graph shows quantification of the percentage of the BrdU-positive population that did not label with Ki67 antibodies (green data points) and the percentage of the Ki-67-positive population that did not label with BrdU antibodies (red data points). (**D**) Confocal image of an adult mouse utricle infected with Ad.MT58A (1×10^9^ TU/mL) that was fixed at 7 DPV and co-labeled with antibodies to PH3-Ser10 (white) and Ki-67 (red). (**E**) Confocal images of a utricle from an embryonic day 17.5 (E17.5) mouse that was fixed *in vivo* and co-labeled with antibodies to PH3-Ser10 (white) and Ki-67 (red). Phalloidin labeling (grayscale) is shown to aid in visualizing the borders of the sensory epithelium. White dashed lines demarcate the borders of the sensory epithelium, and arrows in D and E indicate PH3-Ser10/Ki-67 co-labeled nuclei. Scale bar for D–E, 100 µm. (**F**) Graph shows quantification of the percentage of the Ki-67-positive populations that labeled with antibodies to PH3-Ser10. The difference in the percentage of PH3-Ser10-positive/Ki-67-positive nuclei did not reach statistical significance (p>0.05; Student's t-test). The numbers above the gray bars indicate the mean number of PH3-Ser10-labeled nuclei per sensory epithelium. There were significantly more PH3-Ser10-positive nuclei in E17.5 utricles (p<0.05; Student's t-test).

Similar to co-infected utricles, the mean number of BrdU-labeled nuclei per sensory epithelium in Ad.MT58A-infected utricles increased moderately from 5 to 7 DPV, and then declined by 47% between 7 and 10 DPV (Figs. 3C, 6A–B; mean BrdU-positive supporting cells at 7 DPV  = 414±54, mean BrdU-positive supporting cells at 10 DPV  = 228±44; n = 4 utricles per culture period). The mean number of Ki-67-labeled nuclei per sensory epithelium exhibited a similar temporal pattern, but the percent decrease (34%) was smaller between 7 and 10 DPV (Fig. 6A–B).

Analysis of the percentage of Ki-67-positive supporting cells that did not label with antibodies to BrdU showed that cells in G1 accumulated from 5 to 7 DPV, and then many entered S-phase, exited the cell cycle, or died between 7 and 10 DPV (Fig. 6C; percentage of Ki-67-positive cells that were negative for BrdU at 7 DPV  = 47±7%; percentage of Ki-67-positive cells that were negative for BrdU at 10 DPV  = 30±7%). We also analyzed the percentage of the BrdU-positive population that did not label with antibodies to Ki-67 and found a steady increase from 48±8% at 5 DPV to 60±5% at 10 DPV, indicating cells progressively exited the cell cycle with increased time in culture (Fig. 6C). Regression analysis showed the increase was linear with an exit rate of 2.3% per day (r^2^ = 0.94).

The nuclei of cells that have just completed cytokinesis are typically smaller than nuclei in later phases of the cell cycle [49], and the majority of BrdU-positive/Ki-67-negative nuclei appeared smaller than their BrdU-positive/Ki67-positive counterparts (arrows in Fig. 6A). To further assess whether most cycling cells were proceeding to mitosis, we fixed other cultures at 7 DPV and co-labeled them with antibodies to phosphorylated serine 10 on histone H3 (PH3-Ser10) and Ki-67. Antibodies against PH3-Ser10 recognize chromatin condensation of mitotic cells in M phase [50]. Quantification showed the mean number of PH3-Ser10-labeled supporting cells and the percentage of the Ki-67-positive population that was also PH3-Ser10-positive were both low (Fig. 6D, F; mean PH3-Ser10-positive nuclei per sensory epithelium  = 5±1, percentage of PH3-Ser10-positive/Ki-67-positive nuclei  = 1.7±0.4%; n = 6 utricles). Preliminary analysis showed that the numbers of PH3-Ser10-positive nuclei were similar at 5, 6, and 8 DPV (n = 2 utricles per culture duration, data not shown). While these data do not rule out that progression to M phase may be inefficient in Ad.MT58A-infected utricles, they indicate that at least some supporting cells that reenter the cell cycle can proceed to mitosis.

### The percentage of cells in M phase is similar in Ad.MT58A-infected utricles and developing utricles from embryonic mice

M phase is typically the most rapid phase of the cell cycle [51]–[53], so immunocytochemisty with antibodies to PH3-Ser10 may detect few mitotic cells, particularly if a population of proliferating cells is not cell cycle synchronized. Thus, we sought to determine how the percentage of mitotic supporting cells in Ad.MT58A-infected utricles compared to those in the sensory epithelium of embryonic utricles developing *in vivo*. For this, we fixed utricles from embryonic day 17.5 (E17.5) mice and labeled them with antibodies to Ki-67 and PH3-Ser10 to quantify the number of actively cycling cells in M phase within the sensory epithelium at the time of fixation. Terminal mitoses and expansion of the utricular sensory epithelium in mice do not cease until several days after birth, and significant numbers of cycling cells can still be detected at E17.5 [4], [19].

The mean number of PH3-Ser10-positive nuclei in the E17.5 sensory epithelium was significantly higher than in utricles infected with Ad.MT58A (Fig. 6E–F; mean PH3-Ser10-positive cells  = 10±1; p<0.05, Student's t-test; n = 4 utricles). However, the percentage of the actively cycling population in M phase was comparable to that in Ad.MT58A-infected utricles at 7 DPV (Fig. 6E–F; percentage of PH3-Ser10-positive/Ki-67-positive nuclei in E17.5 utricles  = 1.0±0.1%; p>0.05, Student's t-test). The similar percentages suggest that Ad.MT58A infection may result in significant levels of cell division. A study examining cyclin D1 overexpression in adult mouse utricles found a similarly low percentage of PH3-Ser10-positive/Ki-67-positive supporting cells and concluded that M phase progression was rare and inefficient [39]. Precisely quantifying M phase progression efficiency may be difficult, and cell cycle progression may be different *in vitro* compared to the utricle's natural environment *in vivo*; therefore, the ultimate determination of the ability of these methods to stimulate significant cell replacement may have to wait for tests of recovery in cell number using an *in vivo* damage model.

### A portion of the supporting cells that reenter the cell cycle remain viable

**Figure 7:**
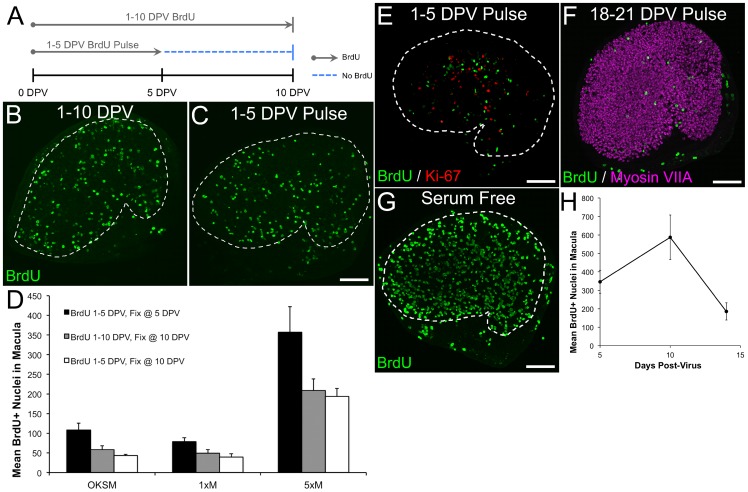
Some supporting cells in Ad.MT58A-infected utricles survive for weeks in culture after reentering the cell cycle. (**A**) Diagram depicting the BrdU labeling paradigm. BrdU was either included in the culture medium for the entire culture period after infection with adenovirus, or it was washed out at 5 days post-virus (DPV) and utricles were cultured for an additional 5 d in its absence. (**B–C**) Confocal images show Ad.MT58A-infected utricles (1×10^9^ TU/mL) fixed at 10 DPV after being cultured with BrdU from 1–10 DPV (B) or 1–5 DPV (C). Scale bar for B–C, 100 µm. (**D**) Graph shows quantification of the number of BrdU-labeled cells in the sensory epithelium from utricles cultured as depicted in A (gray and white bars). Quantification of 5 DPV BrdU labeling (same as in Fig. 5G) is shown to visualize the decline in BrdU-labeled cells from 5 DPV (black bars) to 10 DPV. Data shown is from co-infection experiments (OKSM), infection with 2×10^8^ TU/mL Ad.MT58A (1×M), and infection with 1×10^9^ TU/mL Ad.MT58A (5×M). (**E**) Confocal image of an Ad.MT58A-infected utricle (1×10^9^ TU/mL) fixed at 21 DPV after being cultured with BrdU from 1–5 DPV. Antibody labeling for BrdU and Ki-67 is shown in green and red, respectively. Scale bar, 100 µm. (**F**) Confocal image of an Ad.MT58A-infected utricle (1×10^9^ TU/mL) fixed at 21 DPV after being cultured with BrdU from 18–21 DPV. Antibody labeling for BrdU and myosin VIIA is shown in green and magenta, respectively. Scale bar, 100 µm. (**G**) Confocal image of an Ad.MT58A-infected utricle fixed at 10 DPV after switching from growth medium to differentiation medium at 5 DPV. BrdU (green) was included in the medium throughout. Scale bar, 100 µm. (**H**) Graph shows the mean number of BrdU-positive nuclei per sensory epithelium at 5, 10, and 14 DPV for the experiment described in G. White dashed lines demarcate the borders of the sensory epithelium in all panels.

Knock-down of pocket proteins and cyclin dependent kinase inhibitors that limit cell cycle progression have been shown to stimulate division of hair cells and supporting cells; however, the progeny of these divisions rapidly die and can disrupt the integrity of the sensory epithelium [54]–[62]. Since many proliferating supporting cells underwent apoptosis after Ad.MT58A infection (Figs. 3C, 6B), we sought to determine whether the BrdU-labeled cells detected at 10 DPV were cells that had just recently entered S-phase or whether they replicated their DNA early in the culture and then survived. When we cultured Ad.MT58A-infected utricles (1×10^9^ TU/mL) with BrdU for the first 5 DPV, washed it out, and then cultured for 5 more days (i.e. to 10 DPV) in its absence, the mean number of BrdU-labeled nuclei per sensory epithelium was lower than in utricles subjected to continuous BrdU labeling for all 10 DPV (Fig. 7A–D; mean BrdU-positive nuclei per sensory epithelium at 10 DPV after 1–5 DPV BrdU pulse  = 194±21; n = 4 utricles); however, this difference failed to reach significance (p>0.05, Student's t-test). Similar trends were observed when we used the same pulse-labeling regimen on cultures co-infected with Ad.O (5×10^7^ TU/mL), Ad.K (2×10^8^ TU/mL), Ad.S (5×10^7^ TU/mL), and Ad.MT58A (2×10^8^ TU/mL) or infected with the lower concentration of just Ad.MT58A (2×10^8^ TU/mL; Fig. 7D).

The percent difference between the number of BrdU-labeled cells in utricles fixed at 5 DPV after continuous BrdU labeling and in utricles fixed at 10 DPV after the 1–5 DPV BrdU pulse was 44%, which indicates that 66% of cells entering S-phase prior to 5 DPV are able to survive out to 10 DPV. Furthermore, some of the surviving cells persisted for several weeks since we still detected BrdU-labeled nuclei in the sensory epithelium when we pulse labeled with BrdU from 1–5 DPV and waited to fix the cultures until 21 DPV (Fig. 7E).

To determine whether supporting cells in Ad.MT58A-infected utricles retained the ability to enter S-phase after extended culture, we pulse labeled with BrdU from 18–21 DPV and fixed the cultures at 21 DPV. Labeling with antibodies to BrdU and Ki-67 revealed that supporting cells were still actively cycling and entering S-phase during this period (Fig. 7F). Together, the results suggest that a portion of the cells that reenter the cell cycle after Ad.MT58A infection remain viable, and some retain the ability to enter S-phase for at least 3 weeks in culture.

### BrdU-labeled cells with hair-cell-like characteristics appear after culturing Ad.MT58A-infected utricles in serum free medium

Exchanging growth medium for differentiation medium can promote the differentiation of otic progenitors into hair cells [31], [63], [64]. Differentiation medium is serum-free, however, and c-Myc-expressing cells can enter apoptosis after withdrawal of growth factors [65]. Thus, we were surprised when we saw an increase in the number of BrdU-labeled cells in Ad.MT58A-inected utricles (1×10^9^ TU/mL) after we exchanged growth medium for differentiation medium at 5 DPV and fixed the cultures at 10 DPV (Fig. 7G–H; mean BrdU-positive nuclei per sensory epithelium at 10 DPV  = 588±121; n = 4 utricles). This could possibly be attributed to the T58A mutant, which reduces the sensitivity of c-MycT58A-expressing cells to apoptosis induced by serum deprivation [42], [43], [45]. The increase in the number of BrdU-labeled cells was only temporary, however, as the levels of labeled cells decreased 68% between 10 and 14 DPV (Fig. 7H; mean BrdU-positive nuclei per sensory epithelium at 14 DPV  = 186±47 BrdU-positive nuclei; n = 4 utricles). The reasons for this temporary delay in cell death remain unclear, but serum deprivation lengthens G1 in murine fibroblasts overexpressing c-Myc, so switching to serum-free differentiation medium could have slowed cell cycle progression and the onset of apoptosis [66].

Since serum removal temporarily delayed cell death and led to an increase in the number of BrdU-labeled nuclei, we wondered whether the accumulation of cells that were potentially dividing would be accompanied by expansion of the sensory epithelium. Using myosin VIIA labeling to delineate the sensory epithelium, we made measurements of the macular area in Ad.MT58A- or Ad.GFP-infected utricles (1×10^9^ TU/mL) cultured in differentiation medium from 5–10 DPV. Infection with Ad.MT58A led to very modest, but significant expansion of the sensory epithelium (mean macular area of Ad.MT58A-infected utricles  = 0.19±0.01 mm^2^, mean macular area of Ad.GFP-infected utricles  = 0.17±0.01 mm^2^; p = 0.007, Student's t-test; n = 11 utricles), which further supported the hypothesis that significant numbers of supporting cells in Ad.MT58A-infected utricles are able to progress to M phase and divide.

**Figure 8:**
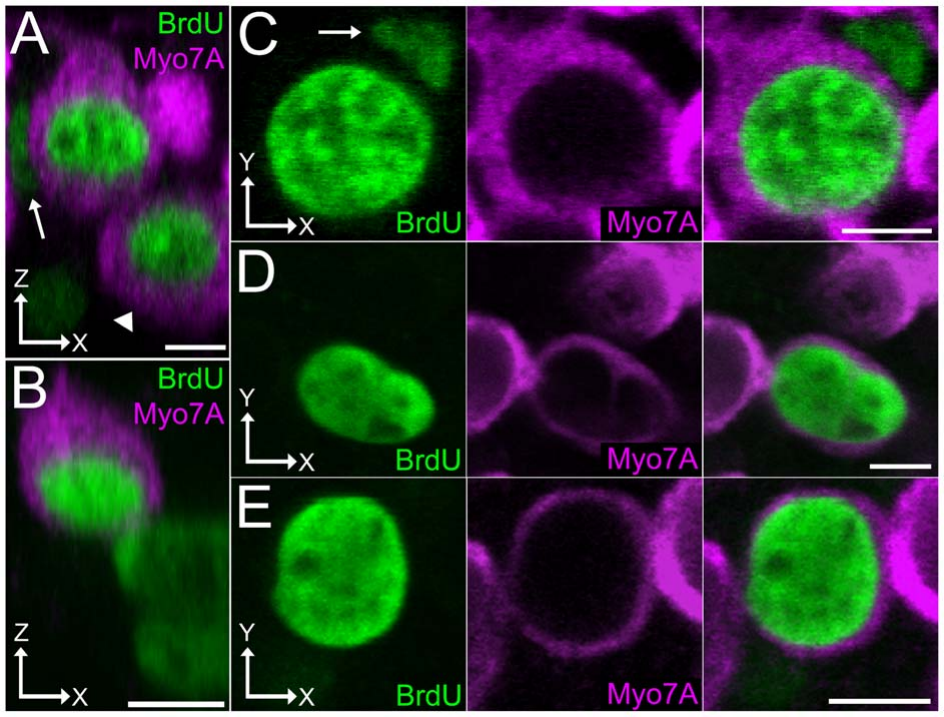
Supporting cells that reenter the cell cycle in Ad.MT58A-infected utricles may be capable of differentiating towards a hair-cell-like fate. Shown are confocal images taken from Ad.MT58A-infected utricles (1×10^9^ TU/mL) fixed at 10 days post-virus (DPV) and labeled with antibodies to BrdU (green) and myosin VIIA (magenta). Growth medium was exchanged for differentiation medium at 5 DPV. (**A–B**) Confocal images show views parallel to the long axis of the hair cells. The arrowhead points to a BrdU-positive/myosin-VIIA-positive cell that does not extend to the apical surface and is probably damaged or dying. The other BrdU-positive/myosin-VIIA-positive cells extend from the hair nuclear layer up to the apical surface, where they appear to display tiny, bundle-like projections. BrdU-positive/myosin-VIIA-positive cells are located within close proximity to these hair-cell-like cells (arrows), suggesting a divisional pair. Scale bars, 5 µm. (**C–E**) Confocal images of three hair-cell-like cells at the level of the hair cell nuclear layer. Arrow in C indicates a neighboring cell that is BrdU-positive/myosin-VIIA-negative. Scale bars, 5 µm.

Although we never detected BrdU-labeled cells that also labeled with hair cell markers such as myosin VIIA in Ad.MT58A-infected utricles cultured with growth medium, Ad.MT58-infected utricles cultured with differentiation medium from 5–10 DPV contained a small number of BrdU-positive/myosin VIIA-positive cells (multiple examples shown in Fig. 8; range of 1–4 BrdU-positive/myosin VIIA-positive cells per utricle; n = 4 utricles). These cells had a chalice shape typical of hair cells, lacked connections with the basal lamina, and extended from the apical surface to the hair cell nuclear layer. Some were rounded and did not extend to the apical surface, suggesting they were damaged or dead (arrowhead in Fig. 8A). Most were paired with a BrdU-positive nucleus that did not label for myosin VIIA (arrows in Fig. 8A, B), suggesting a supporting cell had divided and one of the progeny had become a new hair cell. None displayed a prototypical, F-actin-rich hair bundle as determined with fluorescent phalloidin labeling, indicating these cells were not fully differentiated hair cells with functional mechanotransduction apparatuses. Myosin VIIA is present in stereocilia, however, and small projections from the apical surface could be seen with myosin VIIA-labeling, suggesting that hair bundles may have been in the very early phases of formation (Fig. 8A, B). Since we observed a small number of GFP-expressing hair cells after infection with Ad.GFP (Fig. 1G–H), we cannot rule out that the BrdU-positive/myosin VIIA-positive cells were preexisting hair cells that reentered the cell cycle after being transduced with Ad.MT58A. However, this appears unlikely since we never observed such cells in Ad.MT58-infected utricles cultured with just growth medium. Also, we observed 1–3 BrdU-positive nuclei in the Ad.GFP-infected control utricles that we cultured with differentiation medium from 5–10 DPV, but none of these cells were myosin VIIA-positive. The results suggest that at least a small portion of supporting cells within intact vestibular organs may retain competency for differentiating into hair-cell-like cells after Ad.MT58A-induced cell cycle reentry.

## Discussion

Here we examined how adenoviral delivery of the four iPSC transcription factors to adult mouse utricles affects regenerative potential within the sensory epithelium *in vitro*. We found that adenovectors encoding the T58A variant of c-Myc can initiate cell cycle reentry of postmitotic supporting cells. A portion of the cells that reenter the cell cycle survive for weeks in culture, proceed to mitosis, and appear to express the hair cell marker myosin VIIA under differentiating culture conditions. Cell cycle reentry also corresponded with a very modest, but significant expansion of the sensory epithelium, suggesting that ectopic expression of *MYC* genes may be capable of stimulating regrowth of the sensory epithelium after cells have been lost to damage.

### Suppression of *MYC* may limit the proliferative potential of supporting cells in adult mammals

Although we observed low numbers of supporting cells ectopically expressing c-MycT58A, infection with Ad.MT58A led to robust supporting cell S-phase entry, suggesting that c-Myc levels may be finely balanced within the utricle to suppress proliferation and maintain the postmitotic state. The half-life of c-MycT58A is shorter than that of Oct3/4 and Klf4 in some cell types (Klf4, 120 min in human esophageal cancer cells [67], [68]; Oct3/4, 90 min in mouse embryonic carcinoma cells [69]; and c-MycT58A, 51–63 min in NIH3T3 and REF52 fibroblasts [43], [45]), but the differences in stability do not appear great enough to explain the large differences in the number of supporting cells expressing detectable levels of Oct3/4 and Klf4 compared to c-MycT58A (Fig. 4). This suggests that cells within the sensory epithelium of the adult mouse utricle may have active mechanisms for suppressing c-Myc protein levels, most likely at the post-transcriptional level since the CMV promoter efficiently drives gene expression in supporting cells (Figs. 1,4).

Because there were few cells that labeled with antibodies to c-Myc and HA, we were not able to determine whether Ad.MT58A infection induced proliferation through autonomous or non-autonomous effects. Given c-Myc's well-documented role in directly regulating cell cycle machinery [70], it seems unlikely that supporting cell proliferation was stimulated by paracrine signaling from neighboring cells that were transduced by Ad.MT58A, especially since we did not detect any c-Myc-labeled nuclei in the sensory epithelium of Ad.GFP-infected controls. However, c-Myc overexpression has recently been shown to down-regulate the secretion of proteins that inhibit proliferation in a nontransformed epithelial cell line [71]. Therefore, uncovering the mechanism by which Ad.MT58A drives proliferation could reveal novel insights into how the sensory epithelium maintains the postmitotic state, and it will be essential for determining whether c-Myc can reprogram supporting cells into otic progenitors.

Upstream signals that might limit c-Myc translation or promote its degradation in supporting cells have not been identified, but F-actin and the tumor suppressor E-cadherin accumulate at mammalian supporting-cell-supporting-cell junctions in coordination with the postnatal decline in regenerative capacity, and it has been posited that the signals and molecules responsible for junctional reinforcement may somehow limit regeneration [4], [17], [18], [72]. In epithelial cells, c-Myc specifically activates or represses E-cadherin depending on the expression levels of the two proteins encoded by *MYC*, c-Myc1 and c-Myc2, and increased E-cadherin expression in response to increased c-Myc levels can prevent cellular transformation [73], [74]. This sensitive feedback loop could potentially explain the concomitant decline in proliferation and accumulation of E-cadherin in mammalian supporting cells. c-Myc is a component of many signaling networks, having been estimated to regulate ∼30% of genes [75], so potential repressors of c-Myc are numerous.

### *MYC* gene family members are potential targets for stimulating cell replacement in the mammalian inner ear

The supporting cell proliferation we observed in adult mouse utricles after Ad.MT58A infection suggests that targeted upregulation of c-MycT58A or possibly even wild-type c-Myc may be a viable strategy for stimulating cell replacement in mammalian inner ear sensory epithelia. The *MYC* gene family members, which in mammals include the oncogenes *MYC* (c-Myc), *MYCL* (L-Myc), and *MYCN* (N-Myc), are basic Helix-Loop-Helix Leucine Zipper transcription factors that play a prominent role in regulating cell proliferation, growth, apoptosis, metabolism, and differentiation [65], [70], [76]. Despite the robust proliferative response we observed after Ad.MT58A infection, conditional deletion of c-Myc in the embryonic mouse inner ear has no phenotype, while deletion of N-Myc reduces proliferative growth and disturbs morphogenesis [77], [78]. Some proliferation was still detected in N-Myc mouse mutants, and it remains unclear whether this was due to compensation by another *MYC* family member like c-Myc. These results suggest N-Myc could play a more prominent role in controlling proliferative regeneration in the mammalian inner ear, and overexpression of N-Myc in adults could be more efficient at inducing cell proliferation and promoting survival and differentiation into the correct cell types.

*MYC* can be a potent oncogene, so *MYC* gene therapy may not be a realistic approach for stimulating regeneration in humans [79]. In addition, c-MycT58A is more efficient than wild-type c-Myc at inducing immortalization and transformation [80], [81], which increases the probability that some cells in our cultures were being immortalized or transformed. In support of this possibility, we observed cells actively in the cell cycle and entering S-phase after 3 weeks in culture (Fig. 7). Consequently, small molecules that target *MYC* and its binding partners may be more useful for a therapy that can be used in humans.

### Similarities and differences between proliferation induced by cyclin D1 and c-MycT58A

Adenovector-mediated expression of cyclin D1 (Ad.CD1) in cultured utricles from adult mice robustly induces supporting cell S-phase entry [39], [41]. At 7 d after Ad.CD1 infection, 0.6% of the actively cycling cells (i.e. Ki-67-positive) were in M-phase (i.e PH3-Ser10-positive). This percentage was ∼4-times lower than in P9 mouse utricles infected with Ad.CD1, and it was ∼3-times lower than the percentage we observed in Ad.MT58A-infected utricles from adults (Fig. 6). A thorough analysis, which showed DNA damage in Ki-67-positive cells, minimal Aurora B kinase antibody labeling, few cells in cytokinesis, few cells completing multiple rounds of division, and no hyperplasia of the sensory epithelium led to the conclusion that ectopic cyclin D1 expression in supporting cells was inefficient at inducing cell cycle progression to mitosis.

Determining the exact percentage of cells capable of proceeding to M-phase may be difficult, especially if the proliferating population is not cell cycle synchronized. We detected similar numbers of PH3-Ser10-positive cells at 5, 6, 7, and 8 DPV, which suggests S-phase entry after Ad.MT58A infection is stochastic. Mitosis is typically the shortest phase of the cell cycle, making cells in M-phase the smallest fraction of the cycling population, and we detected low numbers of mitotic cells in both embryonic utricles from developing mice and Ad.MT58A-infected utricles from adult mice. While the percentage of cells in M phase was higher in embryonic and Ad.MT58A-infected utricles compared to Ad.CD1-infected utricles, the percentages in all cases were relatively low, underscoring the need for further characterization of cell cycle progression under all these conditions. It should be noted that many supporting cells are exiting the cell cycle around E17.5 in the utricle [19], and in the rat retina, cell cycle length increases as more cells become postmitotic [82]. Therefore, it is possible that a significantly higher percentage of M phase cells may be present in the utricle at timepoints earlier than E17.5. In addition, ectopic c-Myc expression shortens the duration of G1 phase in murine fibroblasts and reduces the synchronization of cell cycle entry, consistent with our findings here [66]. The shortened G1 phase duration could increase the percentage of cells in M phase, potentially explaining why we found a higher percentage of M phase cells in utricles infected with Ad.MT58A compared to Ad.CD1.

### Can ectopic c-MycT58A expression be used to reprogram supporting cells into multipotent otic progenitors?

The appearance of a small number of BrdU-positive/myosin-VIIA-positive cells after culturing in differentiation medium suggests that at least some supporting cells that reenter the cell cycle after Ad.MT58A infection retain otic identity and competency for differentiating into hair-cell-like cells. The paucity of these cells could be attributed to the inability of the culture environment to replicate induction cues that may be present *in vivo*. Consistent with this notion, proliferation induced by hair cell death in newborn mouse utricles gave rise to new hair cells *in vivo* but not *in vitro* [4]. Alternatively, we did observe a small number of hair cells that were transduced with Ad.GFP, and we have not yet ruled out that the BrdU-positive/myosin VIIA-positive cells were pre-existing hair cells that reentered the cell cycle after being transduced with Ad.MT58A. Conditional deletion of the retinoblastoma protein or ectopic expression of the HPV-16 E7 oncogene within hair cells can drive their reentry into the cell cycle, so it is reasonable to suspect that ectopic expression of c-Myc may produce similar effects [55]–[57], [59], [62], [83]. If the BrdU-positive/myosin VIIA-positive cells we observed did originate from the supporting cell population infected with Ad.MT58A, then ectopic c-Myc expression may be capable of dedifferentiating supporting cells into cells with characteristics of otic progenitors.

The effects of Oct3/4, Klf4, and Sox2 are not observed until later in the iPSC reprogramming process [21], and these factors did not appear to influence proliferation over the time periods we investigated. Expression of all four factors within individual supporting cells may have been unlikely since each was encoded by a separate adenovirus, and it remains to be determined whether simultaneous expression of Oct3/4, Klf4, and Sox2 would aid in directly reprogramming supporting cells into otic progenitors.

Transient expression of the four iPSC transcription factors under cardiac- or neural-promoting culture conditions directly reprograms fibroblasts into cardiomyocytes or neural stem/progenitor cells, respectively [24], [25]. This short expression appears to induce epigenetic activation that results in an unstable, partially reprogrammed state that bypasses pluripotency but remains amenable to differentiation [84]. Similarly, we hypothesize that when the four iPSC factors are delivered to terminally differentiated somatic cells *in situ*, the native organ environment may be able to suppress complete reprogramming to induced pluripotency while allowing direct reprogramming into lineage-restricted progenitor/stem cells. Lineage-specific transcription factors have been used to transdifferentiate cardiac fibroblasts into cardiomyoctes within intact mouse hearts *in vivo*, which demonstrates the feasibility of *in situ* reprogramming [85].

Furthermore, the bHLH transcription factor Atoh1 is both necessary and sufficient for hair cell differentiation during development, and ectopic expression of Atoh1 *in situ* appears to directly reprogram inner ear epithelial cells into hair cells in some instances [86]–[93]. Directly reprogramming postmitotic supporting cells into hair cells could decrease the size of the supporting cell population without some form of nonautonomous cell replacement [92], which makes restoration of self-renewal capacity vital to the reprogramming strategy. Cyclins, cyclin dependent kinase inhibitors (CDKIs), and pocket proteins have been shown to regulate proliferation and cell cycle exit during development of the sensory epithelium, and a growing list of these genes have been targeted to force reentry of supporting cells into the cell cycle [41], [54]–[62], [83], [94]–[98]. *MYC* controls the expression and activity of various cyclins, CDKIs, and pocket proteins [65], [76], which may make it more suitable for orchestrating the complex fluctuations of cell cycle proteins that are necessary to drive efficient progression through the various restriction points. If *MYC* alone is unable to reprogram supporting cells into true otic progenitors, then combining forced cell cycle reentry – either via a *MYC* family member or direct targeting of cell cycle machinery – with ectopic Atoh1 expression may be a more successful cocktail.

In summary, ectopic *MYC* expression shows promise for restoring proliferative capacity to inner ear sensory epithelia and may be used to stimulate regeneration. Further investigations should test this potential by characterizing supporting cell genotype, phenotype, transformation, survival, and differentiation after ectopic c-MycT58A expression in both auditory and vestibular organs *in vivo*.
